# Supplementary material for: Sociodemographic characteristics, community engagement and stigma among Men who have Sex with Men (MSM) who attend MSM-led versus public sexual health clinics: A cross-sectional survey in China
Source: PLoS One. 2024 Oct 16;19(10):e0310957. doi: 10.1371/journal.pone.0310957 (PMC11482664; doi:10.1371/journal.pone.0310957)
Supplement: S1 File — (PDF) [file pone.0310957.s002.pdf]

A. Sociodemographics
The next set of questions will ask you to provide some information about yourself.

A1. Age: ____ years old

A2. Current marital status:
1. Never married
2. Engaged or Married
3. Separated or divorced
4. Widowed

A3. Highest level of completed education:
1. Elementary
2. Middle school
3. High school or vocational school
4. Bachelor or associate degree
5. Above bachelor's degree

A4. Have you ever volunteered in the past? By volunteer, we mean providing unpaid service to one or more people.
1. Yes
2. No
3. Unsure

A5. Have you ever volunteered for MSM-related causes, events, or organizations?
1. Yes
2. No
3. Unsure

A6. What is your total individual **monthly** income from all sources?
1. <1500 RMB/month
2. 1500-3000 RMB/month
3. 3001-5000 RMB/month
4. 5001-8000 RMB/month
5. >8000 RMB/month

A7. Who did you have sex with in the past 12 months? Sex here refers to oral, anal, or vaginal intercourse.
1. I have not had sex in the past 12 months
2. Only men
3. Only women (skip to J1)
4. Both men and women
5. Prefer not to say

MSM PARTICIPANTS

B. Sexual behaviors
The next set of questions will ask you about your sexual behaviors with other men.

B1. What is your role during anal sex?
1. Mostly receptive (bottom)
2. Mostly insertive (top)
3. Half and half (versatile)

B2. In the past 3 months, how many male sexual partners have you had?
(Number) partners

B3. In the past 3 months, when you had anal sex, how frequently did you use condoms?
1. I did not have anal sex
2. 0% condom use
3. Less than 50% condom use
4. More than 50% condom use
5. 100% condom use

B4. In the past 3 months, when you had vaginal sex, how frequently did you use condoms?
1. I did not have vaginal sex
2. 0% condom use
3. Less than 50% condom use
4. More than 50% condom use
5. 100% condom use

B5. In the past 3 months, when you had oral sex, how frequently did you use condoms?
1. I did not have vaginal sex
2. 0% condom use
3. Less than 50% condom use
4. More than 50% condom use
5. 100% condom use

B6. In the past, have you told anyone about your sexuality or sexual history with men? (Select all that apply)
1. Yes, my long-term female partner/wife
2. Yes, my family members
3. Yes, my friends
4. Yes, my healthcare providers
5. Yes, others:_________
6. No one

B7. How do you identify in terms of your sexual orientation?
1. Gay
2. Bisexual
3. Heterosexual
4. Unsure
5. Another sexual orientation, please specify

B8. Would you say that you are open (out) as gay, bisexual, or a man attracted to other men?
1. Not at all open (out)
2. A little bit open (out)
3. Somewhat open (out)
4. Very Much open (out)
5. Open (out) to all or most people you know

C. Clinical Information

C1. Do you have any symptoms that you are worried may be due to an STI?
1. Yes. Symptoms: _____________
2. No

C2. Have you ever tested for HIV in the past?
1. Yes
2. No

C3. Before today, have you ever tested for gonorrhea in the past?
1. Yes
2. No

C4. Before today, have you ever tested for chlamydia in the past?
1. Yes
2. No

C5. Today, did you agree to get tested for gonorrhea and chlamydia?
1. Yes
2. No

D. Community Engagement

The next set of questions asks about your experiences with MSM-related causes, events and organizations in your community.

D1. Have you ever participated in online forums or discussions on social media (ie. Weixin, Weibo, Twitter, or other on-line communities) about issues related to the MSM community?

o Yes
o No

D2. Are you aware of any ongoing MSM-related community events?

o Yes
o No

D3. Have you ever encouraged someone to use MSM-related community resources, such as free HIV and syphilis testing services?

o Yes
o No

D4. Have you ever attended MSM-related community events?
o Yes
o No

D5. Have you ever donated to MSM-related causes, events, or organizations? (other than today)
o Yes
o No

E. Community Connectedness
The following set of questions asks about your feelings toward the MSM. Here, "MSM community" broadly refers to the collective of individuals and community organizations that have an interest in MSM-related issues.

E1. You feel that you are a part of the MSM community.
o Strongly Agree
o Agree
o Disagree
o Strongly Disagree

E2. Participating in the MSM community is a positive thing for you.
o Strongly Agree
o Agree
o Disagree
o Strongly Disagree

E3. You are proud of the MSM community.
o Strongly Agree
o Agree
o Disagree
o Strongly Disagree

E4. It is important for you to be an advocate for the MSM community.
o Strongly Agree
o Agree
o Disagree
o Strongly Disagree

E5. If you and your peers work together, the problems in the MSM community can be solved.
o Strongly Agree
o Agree
o Disagree
o Strongly Disagree

E6. You really feel that any problems faced by the MSM community are also your own problems.
o Strongly Agree
o Agree
o Disagree
o Strongly Disagree

E7. The diagram below is designed to represent your relationship ("Self") with LGBT as a group ("LGBT"). Please indicate your relationship by selecting the option that best captures your relationship with this LGBT as a group.

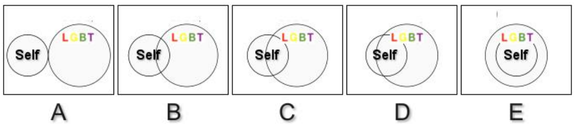

F. Social Cohesion
F1. You can count on other MSM in your group of friends if you need to borrow money.
o Strongly Agree
o Agree
o Disagree
o Strongly Disagree

F2. You can count on other MSM in your group of friends if you need to talk about your problems.
o Strongly Agree

- Agree
- Disagree
- Strongly Disagree

F3. You can count on other MSM in your group of friends if you need somewhere to stay.
- Strongly Agree
- Agree
- Disagree
- Strongly Disagree

F4. The group of MSM with whom you socialize with is an integrated group.
- Strongly Agree
- Agree
- Disagree
- Strongly Disagree

F5. You can trust the majority of the MSM you know.
- Strongly Agree
- Agree
- Disagree
- Strongly Disagree

F6. In general, MSM in your group of friends in the area where you live only worry about themselves
- Strongly Agree
- Agree
- Disagree
- Strongly Disagree

F7. In general the MSM you socialize with are always arguing amongst each other
- Strongly Agree
- Agree
- Disagree
- Strongly Disagree

F8. If someone offered you the change to be completely heterosexual, you would accept that chance
- Strongly Agree
- Agree
- Disagree
- Strongly Disagree

F9. You have concealed your sexual orientation by avoiding contact with other LGBTQ individuals.
- Not at all
- A little bit
- Somewhat
- Very Much
- All the time

F10. Bystander effect- You have remained silent while witnessing anti-gay remarks, jokes, or activities because you did not want to be labeled as LGB by those involved.
- Not at all
- A little bit
- Somewhat
- Very Much
- All the time

F11. You make anti-gay remarks, jokes, or activities because you did not want to be labeled as LGB by those involved.
- Not at all
- A little bit
- Somewhat
- Very Much
- All the time

F12. You enjoy making other people in your MSM community feel better.
- Strongly Agree

- Agree
- Disagree
- Strongly Disagree

G. Pay-It-Forward Participation

G1. Today, you came to testing:

1. By yourself (Skip to G3)
2. Accompanied by someone else

G2. How would you describe your relationship to the person accompanying you?

1. Sex partner
2. MSM peer
3. Non-MSM peer
4. Family
5. Other, specify:____

G3. Did you create a message, postcard, image, or other material to encourage others to receive testing?

G4. Which of the following did you see?

1. Physicial post card
2. Digital post card

Other

G5. Describe how you felt immediately after you knew that someone from the local community cared about you and donated money for your test. [open answer and white board]

G6. What amount did you donate?
(If no, skip the last two questions)

G7. Now lets talk about after you donated your money. Describe how you felt immediately after your donated. [open answer and white board]

G8. Do you agree or disagree with the following statement:I am so thankful for this PIF program.

1 strongly disagree
2 disagree
3 slightly disagree
4 neutral
5 slightly agree
6 agree
7 strongly agree

G9.Do you agree or disagree with the following statement: If I had to list everything that I felt grateful for, it would be a very long list.

1 strongly disagree
2 disagree
3 slightly disagree
4 neutral
5 slightly agree
6 agree
7 strongly agree

G10.Do you agree or disagree with the following statement:I am grateful to the people in the MSM community.

1 strongly disagree
2 disagree
3 slightly disagree
4 neutral
5 slightly agree
6 agree
7 strongly agree

G11.Do you agree or disagree with the following statement:As I get older I find myself more able to appreciate the people, events, and situations that have been part of my life history.

1 strongly disagree
2 disagree
3 slightly disagree
4 neutral
5 slightly agree
6 agree
7 strongly agree

G12.Do you agree or disagree with the following statement: Long amounts of time can go by before I feel grateful to something or someone.
1 strongly disagree
2 disagree
3 slightly disagree
4 neutral
5 slightly agree
6 agree
7 strongly agree

H.Internalized Homophobia[1]

H1.Sometimes I wish I were not gay.
- o Strongly Agree
- o Agree
- o Disagree
- o Strongly Disagree

H2.Most of the time I am glad to be gay.
- o Strongly Agree
- o Agree
- o Disagree
- o Strongly Disagree

H3.If people my age knew my sexual orientation, I'm afraid that many would not want to be my friends.
- o Strongly Agree
- o Agree
- o Disagree
- o Strongly Disagree

H4.If there were a pill to make me straight I would take it.
- o Strongly Agree
- o Agree
- o Disagree
- o Strongly Disagree

H5.I have tried to stop being attracted to men.
- o Strongly Agree
- o Agree
- o Disagree
- o Strongly Disagree

H6.I'd like to get professional help in order to change my sexual orientation.
- o Strongly Agree
- o Agree
- o Disagree
- o Strongly Disagree

H7.Sometimes I wish I could become more sexually attracted to women.
- o Strongly Agree
- o Agree
- o Disagree
- o Strongly Disagree

H8.I feel that being gay is a shortcoming for me.
- o Strongly Agree
- o Agree
- o Disagree
- o Strongly Disagree

H9.Sometimes I feel ashamed of my sexual orientation.
- o Strongly Agree
- o Agree
- o Disagree
- o Strongly Disagree

---

1 https://www.ncbi.nlm.nih.gov/pmc/articles/PMC5560600/

<u>I.Perceived Gay-Related Stigma</u>[2]

I1.Many people believe that gay men have psychological problems.
- o Strongly Agree
- o Agree
- o Disagree
- o Strongly Disagree

I2.Many people do not see gay men as real men.
- o Strongly Agree
- o Agree
- o Disagree
- o Strongly Disagree

I3.Most families would be disappointed to have a gay son.
- o Strongly Agree
- o Agree
- o Disagree
- o Strongly Disagree

I4.Many people think that gay men have HIV and will die of AIDS.
- o Strongly Agree
- o Agree
- o Disagree
- o Strongly Disagree

I5.Many people do not accept same-sex male couples.
- o Strongly Agree
- o Agree
- o Disagree
- o Strongly Disagree

I6.Many people believe that gay men should not raise children.
- o Strongly Agree
- o Agree
- o Disagree
- o Strongly Disagree

I7.Many people believe that gay men should not hug, hold hands, or kiss in public.
- o Strongly Agree
- o Agree
- o Disagree
- o Strongly Disagree

<u>J. Sexual behaviors</u>

The next set of questions will ask you about your sexual behaviors with women.

J1. In the past 3 months, how many female sex partners have you had with? (Number)
____ partners

J2.In the past 3 months, when you had anal sex, how frequently did you use condoms?

6. I did not have anal sex
7. 0% condom use
8. Less than 50% condom use
9. More than 50% condom use
10. 100% condom use

J3. In the past 3 months, when you had vaginal sex, how frequently did you use condoms?

6. I did not have vaginal sex
7. 0% condom use
8. Less than 50% condom use
9. More than 50% condom use
10. 100% condom use

[2] https://www.ncbi.nlm.nih.gov/pmc/articles/PMC5560600/

J4. In the past 3 months, when you had oral sex, how frequently did you use condoms?
6. I did not have vaginal sex
7. 0% condom use
8. Less than 50% condom use
9. More than 50% condom use
10. 100% condom use

J5. How do you typically find your female sexual partners?
1. Brothel
2. Massage parlour
3. Entertainment establishments (e.g. karaoke lounge, bar, pub, nightclub)
4. Mutual friend
5. Online dating applications (e.g. Tinder, OkCupid, Happn, MeetMe, Badoo)
6. Social media
7. Online forums or websites
8. Other, please specify:

K. Clinical information

K1. Do you have any symptoms that you are worried may be due to an STI?
1. Yes. Symptoms: _____________
2. No

K2. Have you ever tested for HIV in the past?
1. Yes
2. No

L.HIV discrimination and stigma[3]

L1.Do you fear that you could contract HIV if you come into contact with the saliva of a person living with HIV?
1.Yes
2.No

L2.Do you agree or disagree with the following statement: I would be ashamed if someone in my family had HIV
1 strongly disagree
2 disagree
3 slightly disagree
4 neutral
5 slightly agree
6 agree
7 strongly agree

L3.In your opinion, are people hesitant to take an HIV test due to fear of people's reaction if the test result is positive for HIV?
1.Yes
2.No

L4.Do people talk badly about people living with or thought to be living with HIV to others?
1.Yes
2.No

L5.Would you buy fresh vegetables from a shopkeeper or vendor if you knew that this person had HIV?
1.Yes
2.No

M.Pay-It-Forward Participation

M1. Today, you came to testing:
1.By yourself (Skip to K3)
2.Accompanied by someone else

M2. How would you describe your relationship to the person accompanying you?
1. Sex partner
2. A friend
3. Family
4. Other, specify:____

[3] https://www.icrw.org/wp-content/uploads/2017/07/STRIVE_stigma-brief-A4.pdf

M3. Did you create a message, postcard, image, or other material to encourage others to receive testing?

M4. Which of the following did you see?
1. Physicial post card
2. Digital post card
3. Other

M5. Describe how you felt immediately after you knew that someone from the local male community cared about you and donated money for your test. [open answer and white board]

M6. What amount did you donate?
(If no, skip the last two questions)

M7. Now lets talk about after you donated your money. Describe how you felt immediately after your donated. [open answer and white board]

M8. Do you agree or disagree with the following statement:I am so thankful for this PIF program.
1 strongly disagree
2 disagree
3 slightly disagree
4 neutral
5 slightly agree
6 agree
7 strongly agree

M9.Do you agree or disagree with the following statement: If I had to list everything that I felt grateful for, it would be a very long list.
1 strongly disagree
2 disagree
3 slightly disagree
4 neutral
5 slightly agree
6 agree
7 strongly agree

M10.Do you agree or disagree with the following statement:I am grateful to the people in the MSM community.
1 strongly disagree
2 disagree
3 slightly disagree
4 neutral
5 slightly agree
6 agree
7 strongly agree

M11.Do you agree or disagree with the following statement:As I get older I find myself more able to appreciate the people, events, and situations that have been part of my life history.
1 strongly disagree
2 disagree
3 slightly disagree
4 neutral
5 slightly agree
6 agree
7 strongly agree

M12.Do you agree or disagree with the following statement: Long amounts of time can go by before I feel grateful to something or someone.
1 strongly disagree
2 disagree
3 slightly disagree
4 neutral
5 slightly agree
6 agree
7 strongly agree

N.Homophobia[4]

[4] https://www.ncbi.nlm.nih.gov/books/NBK573036/box/ch7_f2/?report=objectonly

N1.Do you think that lesbians or gays can influence others to become homosexual?

1.Yes
2.No

N2.Do you think someone could influence you to change your sexual orientation?

1.Yes
2.No

N3.If someone you cared about said to you, "I think I'm lesbian or gay," would you suggest that the person see a therapist?

1.Yes
2.No

O.Masculine Attributes

O1.A real man has a good paying job.

- Strongly Agree
- Agree
- Disagree
- Strongly Disagree

O2.A real man works hard to get ahead.

- Strongly Agree
- Agree
- Disagree
- Strongly Disagree

O3.A real man has sex with a lot of different women.

- Strongly Agree
- Agree
- Disagree
- Strongly Disagree

O4.A real man puts his male friends (bros) before his girlfriends.

- Strongly Agree
- Agree
- Disagree
- Strongly Disagree

P. Social Cohesion

P1. You can count on other men in your group of friends if you need to borrow money.

- Strongly Agree
- Agree
- Disagree
- Strongly Disagree

P2. You can count on other men in your group of friends if you need to talk about your problems.

- Strongly Agree
- Agree
- Disagree
- Strongly Disagree

P3. You can count on other men in your group of friends if you need somewhere to stay.

- Strongly Agree
- Agree
- Disagree
- Strongly Disagree

P4. The group of men with whom you socialize with is an integrated group.

- Strongly Agree
- Agree
- Disagree
- Strongly Disagree

P5. You can trust the majority of the men you know.

- Strongly Agree
- Agree
- Disagree

- Strongly Disagree

P6. In general, men in your group of friends in the area where you live only worry about themselves
- Strongly Agree
- Agree
- Disagree
- Strongly Disagree

P7. In general the men you socialize with are always arguing amongst each other
- Strongly Agree
- Agree
- Disagree
- Strongly Disagree

P8. You enjoy making other people in your men community feel better.
- Strongly Agree
- Agree
- Disagree
- Strongly Disagree
